# Clinical Evidence-Guided Anti-rheumatoid Arthritis Study of Shuji Tablet in Adjuvant-Induced Arthritis Rats and Mechanism Exploration *via* Network Pharmacological Approach

**DOI:** 10.3389/fphar.2021.694507

**Published:** 2021-07-29

**Authors:** Weibo Dai, Jing Yang, Haili Cao, Zhuqiang Wang, Guangru Li, Xiwen Zhong, Weiwen Peng, Chang Chen, Xin Liu, Congyan Zeng, Xianjing Hu

**Affiliations:** ^1^ Pharmacology Laboratory, Zhongshan Hospital, Guangzhou University of Chinese Medicine, Zhongshan, China; ^2^ Guangzhou Xiangxue Pharmaceutical Co., Ltd, Guangzhou, China; ^3^ Centre for Cancer and Inflammation Research, School of Chinese Medicine, Hong Kong Baptist University, Hong Kong, China; ^4^ Biotechnological Institute of Chinese Materia Medical, Jinan University, Guangzhou, China

**Keywords:** Shuji tablet, rheumatoid arthritis, network pharmacology, traditional Chinese medicine, adjuvant-induced arthritis, mechanism

## Abstract

**Background:** Rheumatoid arthritis (RA) is a kind of chronic autoimmune disease with several tissues damaged. Shuji tablet (SJT) is a prescription approved for treating lumbago and leg pain in the clinic. However, the efficacy of SJT against RA is still unknown. This study aims to evaluate the therapeutic effect of SJT on adjuvant-induced arthritis (AIA) rats and explore the mechanism *via* a network pharmacological approach.

**Methods:** AIA rats were treated with SJT for 30 days at the dosages of 3.6, 1.8, and 0.9 g/kg, respectively, and the anti-RA effect was determined by measuring paw swelling, systemic symptoms score, arthritis index, and histopathological change. ELISA assay was used to evaluate the level of inflammatory cytokines in serum. The mechanism exploration and target prediction of SJT against RA were performed *via* a network pharmacological approach.

**Results:** SJT showed excellent alleviation on AIA rats, with evidence of reducing paws swelling, decreasing systemic symptoms score, and arthritis index. Furthermore, SJT significantly reduced the serum cytokines of IL-6, IL-1β, TNF-α in AIA rats. Histopathological examination showed SJT remarkably reduced synovial hyperplasia, cartilage damage, and inflammatory infiltration in the secondary-side paws. According to network pharmacological analysis, 208 candidate compounds and 445 potential targets of SJT were identified, and 4465 RA therapy-related targets were searched out. Subsequently, 292 target genes of SJT were speculated to be associated with RA treatment, among which the top 5 “response values” targets were STAT3, AKT1, JUN, HSP90AA1, TNF. GO and KEGG enrichment analysis suggested that 45 signaling pathways were associating with SJT treating RA. The top 10 signaling pathways were PI3K-Akt, MAPK, AGE-RAGE pathway in diabetic complications, Ras, HIF-1, TNF, Chemokine, IL-17, FoxO, and Rap1.

**Conclusion:** Our experimental study showed that SJT significantly alleviated rheumatoid arthritis of AIA rats. Network pharmacology showed that the key targets of SJT against RA probably were STAT3, AKT1, JUN, HSP90AA1, TNF, and the potential mechanism was associated with modulation on the signaling pathways of PI3K-Akt, MAPK, Ras, AGE-RAGE, HIF-1, TNF, chemokine, IL-17, FoxO, Rap 1. Our study strongly provides evidence for Shuji tablet in RA therapy and would enlarge its application in the clinic.

## Introduction

Rheumatoid arthritis (RA) is a systemic disease with several tissues damaged, such as synovial membrane, cartilage, and bone. The incidence of RA increases along with age (Tuncer et al., 2017). In North America and Europe, 0.5–1% of adults are suffering from RA (Bao et al., 2019). RA patients are usually suffering from a chronic syndrome of pain, joints swelling and morning stiffness, even deformity, causing disability (Pan et al., 2017). Scientists worldwide are seeking effective remedies to treat RA, including developing new drugs and employing traditional ethnologic therapies. Currently, drugs used for RA treatment include glucocorticoids, non-steroidal anti-inflammatory drugs (NSAIDs), and disease-modifying antirheumatic drugs (DMARDs). Series of anti-RA drugs used in the clinic, such as methotrexate, inhibitors of Janus kinase (JAK), and tumor necrosis factor (TNF), would induce nonnegligible side-effects such as cytopenia, transaminase elevation, cardiovascular disease (CV), and gastrointestinal (GI) events (Walsem et al., 2015; Burmester et al., 2017; Chatzidionysiou et al., 2017). Therefore, it is urgently demanded to develop other new drugs with more efficacy and fewer side effects in RA therapy.

RA is a kind of disease induced by multifactorial factors, including genetic, epigenetic, and environmental factors (Scherer et al., 2020). Pathologically, RA patients would harbor multi-features in the clinic, such as disordered body-immunity, dysregulated cytokine networks, and activated osteoclast and chondrocyte (Firestein et al., 2017). Series of signaling pathways were reported to be referred to in the occurrence and development of RA, such as the JAK/STAT pathway which regulates the gene expression of matrix metalloproteinases of inflamed synovial tissues (Malemud et al., 2018), PI3K/AKT/mTOR pathway which regulates the cell cycle, cellular quiescence and proliferation (King et al., 2015; Feng et al., 2018), MAPK pathway which regulates the production of proinflammatory cytokines (Yang et al., 2018; Thalhamer, et al., 2008), and so on. Hence, a good remedy used in RA therapy should involve the modulation of multiple targets and signaling pathways.

Network pharmacology is a newly emerged analytical method *via* integrating information network and pharmacological approach to identify drugs or disease targets from series of databases, and predict the signaling pathways those the drugs probably modulate (Ao et al., 2020). It is a useful method in the complicated mechanism study for multicomponent drugs, by which the “drug-active ingredient-target-disease network” would be clarified, and the multiple-biological processes, -mechanisms, and -signaling pathways would be comprehensively analyzed (Zhou et al., 2020). Traditional Chinese medicine (TCM) has been used for thousands of years in China. It has the feature of synergistic effect combining with several different kinds of herbal medicine (named “Jun-Chen-Zuo-Shi” in Chinese medicine). Understanding how the multiple ingredients in a herbal formula act in synergy, and the regulation of the active ingredients on multiple targets of diseases is an important approach to develop traditional Chinese medicine. Combining the network science with ancient TCM may potentially explore the scientific evidence of herbal formulae on the basis of complex biological systems (Li et al., 2013). “TCM network pharmacology” methodology is integrating Chinese medicine, network science, information science, and experimental science to study TCM systematically and predictably, and is usually considered to be a highly effective approach for predicting potential pathways and targets of drugs, which is now widely used in investigating the pharmacological mechanism and predicting the candidate targets of TCM (Wang et al., 2017).

**FIGURE 1:**
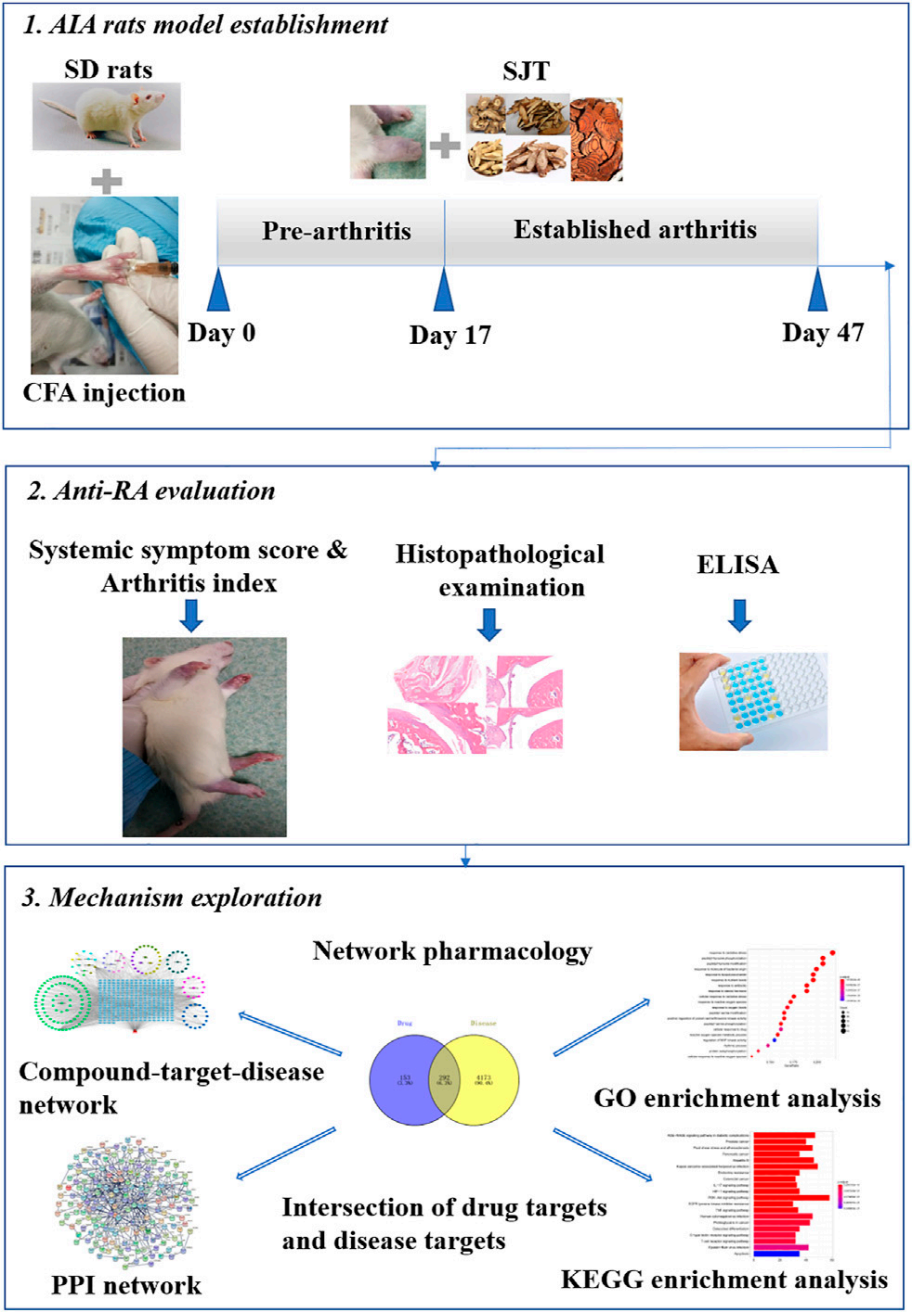
Workflow for the study of SJT on anti-rheumatoid arthritis effect and mechanism exploration *via* network pharmacological approach.

Shuji tablet (SJT), a Chinese medicine prescription, has been approved for treating lumbago and leg pain in the clinic by the Preparation Specification of Medical Institutions in Guangdong Province (approval number Z20130008) (Zeng et al., 2016). SJT formula is composed of 14 medicinal herbs, including *Paeonia lactiflora* Pall. (PL, Baishao), *Ligusticum striatum* DC. (LS, Chuanxiong), *Angelica sinensis* (Oliv.) Diels (AS, Danggui), *Angelica pubescens* Maxim. (AP, Duhuo), *Stephania tetrandra* S. Moore (ST, Fangji), *Smilax glabra* Roxb. (SG, Fuling), *Glycyrrhiza uralensis* Fisch. ex DC. (GU, Gancao), *Spatholobus suberectus* Dunn (SS, Jixueteng), *Callerya speciosa* (Champ. ex Benth.) Schot (CS, Niudali), *Achyranthes bidentata* Blume (AB, Niuxi), *Flemingia prostrata* Roxb. Junior ex Roxb. (FP, Qianjinba), *Taxillus chinensis* (DC.) Danser (TC, Sangjisheng), *Clematis chinensis* Osbeck (CC, Weilingxian), *Tinospora sinensis* (Lour.) Merr. (TS, Kuanjinteng), among which TS can relieve pain induced by chemicals (Cao et al., 2019), and CS can suppress inflammation by decreasing COX-2 (Cao et al., 2019a). According to some literature, the herbs of PL, LS, AS, AP, SG, AB, TC, the major constitutes of Duhuo-Jisheng decoction, were commonly used for RA treatment in the clinic (Zhang et al., 2020), and tetrandrine, one of the main ingredients of ST, was reported to alleviate symptoms of RA through regulating NF-κB and MAPK signaling pathways (Wu et al., 2020). Our previous study had verified the anti-inflammatory and analgesic effects of SJT in rats (Wu et al., 2016). Furthermore, during the clinical practice of treating patients with lumbago or leg pain, SJT was also discovered to relieve RA symptoms in patients. However, the experimental evidence about the efficacy of SJT against RA is still limited, and the mechanism remains unclarified. In our present study, the therapeutic effect of SJT on RA was evaluated *via* setting up adjuvant-induced arthritis (AIA) rat model, and the underlying mechanisms were investigated *via* a network pharmacological approach. The whole study procedure is shown in Figure 1.

## Materials and Methods

### Materials and Regents

All the 14 herbal medicines (PL: 50100; LS: 50078; AS: 50077; AP: 50086; ST:50082; SG: 54201; GU:50012; SS: 54117; CS: 50026; AB: 50090; FP: 50113; TC: 54106; CC: 50029; TS: 54112) contained in SJT were purchased by Kang Sheng Pharmaceutical Company (Guangzhou, China). Tripterygium glycoside (TG) was selected as the positive control and purchased from Huangshi Feiyun Pharmaceutical Co., Ltd. (Huangshi, China). Freeze-dried Bacillus Calmette-Guerin vaccine (BCG) powder used as the complete Freund’s adjuvant (CFA) was purchased from National Vaccine and Serum Institute (Beijing, China). Enzyme-linked immunosorbent assay (ELISA) kits of tumor necrosis factor-α (TNF-α, #RK00029), interleukin-6 (IL-6, #RK00020), interleukin-1β (IL-1β, #RK00009) were purchased from ABclonal Biotech Co., Ltd. (Boston, United States).

### Preparation of SJT

The preparation of SJT in our study was performed following the “SJT Production Standard” recorded in the approved document. Briefly, the powders of PL (45 g), SG (54 g), GU (22.5 g), SS (90 g), CS (135 g), AB (45 g), FP (135 g), TC (67.5 g), CC (45 g), TS (67.5 g) were mixed and decocted twice with deionized water (1:10, w/v) for 2 h each time, then the decoction was filtered. The filtrate was combined and concentrated *via* a rotary evaporator at 60°C. Subsequently, the extract was dried to obtain the powder *via* the spray drying method. The powder was finally mixed completely with the other four pulverized herbals consisting of AP (54 g), LS (45 g), AS (45 g), ST (45 g), to form an SJT prescription. The extraction yield of SJT is 16%, complying with the criterion of Chinese Pharmacopoeia (State Pharmacopoeia Commission., 2020). The SJT powder was stored at 4°C and suspended in distilled water before use.

### UHPLC-QTOF-MS Analysis

SJT powder (0.2 g) was extracted in 8 ml methanol/water (1:1, v/v) for 40 min ultrasonically at room temperature. The extract solution was then centrifuged at 13,000 rpm for 10 min and the supernatant was collected, filtered with a filter (0.22 µm). The sample obtained was applied for components identification through UHPLC-QTOF-MS analysis. Chromatographic separation was performed on a Shimadzu LC-30A liquid chromatograph (Shimadzu, Japan), and the LC conditions were: C18 column (100 mm × 2.1 mm i.d., 1.7 µm; Waters). Water containing 0.1% formic acid (solvent system A) and acetonitrile (solvent system B) served as the mobile phase. The gradient elution program was 0.01–15 min, 5–30% B; 15–35 min, 30–95% B; 35–37 min, 95–95% B; 37–37.1 min, 95–5% B; 37.1–40 min, 5% B. Flow rate: 0.4 ml/min; temperature: 45°C; injection volume: 5 μl. Mass detection was performed using a TripleTOF 5600 (AB Sciex, United States) operating in both positive and negative mode electrospray ionization with the following operating parameters: ion spray voltages for positive and negative modes were 5.5 and −4.5 kV; temperature: 500°C; declustering potential (DP): 100 V; collision energy (CE): ±35 eV. Ion spray and curtain gases were set at 50 and 40 psi, respectively. MS spectra were recorded over the m/z range of 50–1,000 to determine the top six most intense ions for QTOF-MS analysis. All data was processed by Analyst software, version 1.6 TF (Sciex).

### Animals

Sprague Dawley rats (SD, ♂, aged 5–6 weeks) were supplied by Guangdong Medical Laboratory Animal Center [license no. SYXK (Y) 2018-0002]. Rats were maintained in a specific-pathogen-free (SPF) grade animal house with environmental conditions of 23 ± 2°C, 60–70% humidity, 12 h light/dark cycle, and allowed free access to food and water. The animal experiment was performed according to the Laboratory Animal-Guideline for “Ethical Review of Animal Welfare” and approved by the Experimental Animal Management and Ethics Committee of Guangzhou University of Chinese Medicine.

### AIA Rats Model Construction and Drug Administration

CFA was used to set up the AIA model as previously described (Feng et al., 2004). Briefly, CFA was prepared using 150 g freeze-dried BCG powder emulsified in 12 ml light liquid paraffin. SD rats, except for the normal group rats, were inoculated with CFA (0.1 ml/rat) in the right hind paw after receiving inhalational anesthesia *via* a tabletop anesthesia machine (Harvard, United States). The normal group rats were injected with 0.1 ml of normal saline. After 17 days, the rats with systemic symptom scores being more than six were randomly divided into six groups (12 rats/group), including normal saline (control) group, AIA (model) group, positive control (TG, 9.5 mg/kg) group, SJT high dosage (3.6 g/kg) group, SJT middle dosage (1.8 g/kg) group, and SJT low dosage (0.9 g/kg) group. From day 17th onward, SJT and TG were orally administered for 30 days continuously. The normal appearances of rats were monitored and body weights were regularly recorded. The rats were sacrificed after treatment, and the organs including the thymus, spleen were removed and weighed. Organ indexes were calculated according to the formula: organ index (mg/g) = organ weight (mg)/body weight (g).

### Determination for Paw Swelling

Paw thicknesses were measured *via* a vernier caliper purchased from Jingjiang Measuring Tools Co., Ltd. (Jingjiang, China). The thicknesses were measured on day 0 (before CFA injection) and day 17th (before treatment), and regularly monitored (once/6 days) during drugs treatment.

### Evaluation of Arthritis

A systemic symptom score table (Wang et al., 2011) and a 5-point ordinal scale scoring system (Zhang et al., 2008) were used to quantitatively evaluate the severity of arthritis in two dimensions. The systemic symptom score reflected the inflammatory response of the whole body, and each rat could receive 0–8 points. The arthritis index was used to measure the inflammation of three paws (except the right hind paw), and it was graded from 0 to 4, with a maximum score of 12 points per rat.

### ELISA Assay

After drug administration, the blood was collected and serum was isolated after centrifugation at 4,000 rpm for 10 min. Levels of TNF-α, IL-6, and IL-1β in serum were determined by commercial ELISA assay kits. The procedure was conducted according to the manufacturer’s instructions.

### Histopathological Examination

The left hind paws (the secondary side) were removed and fixed in 10% (v/v) neutral formalin after the rats were sacrificed. The ankle joint tissues were put into 10% (w/v) ethylene diamine tetraacetic acid to decalcify for 60 days (Pan et al., 2017). After that, tissue samples were embedded in paraffin and cut into 4 μm sections. Slices were stained with hematoxylin-eosin (HE) to observe the histopathological changes under a light microscope.

### Collection for Chemical Composition and Target Information of SJT

The Traditional Chinese Medicine Systems Pharmacology Database and Analysis Platform (TCMSP) (https://tcmspw.com/tcmsp.php), which recorded 499 common traditional Chinese medicines, was used to collect the chemical composition of SJT. The potential active ingredients in the herbals of SJT were determined according to the information of drug-like (DL) and oral bioavailability (OB), with the selected criteria of DL ≥ 0.18 and OB ≥ 30% in this study. After that, the PubChem database (https://pubchem.ncbi.nlm.nih.gov/) was used to check the compound names and molecular structures. Concurrently, target genes of these active ingredients were predicted by searching the TCMSP database and corrected by the Uniprot database (https://www.uniprot.org/).

### Acquisition of Disease Targets

The “rheumatoid arthritis” was used as a keyword in searching Genecards database (https://www.genecards.org/) and OMIM database (https://omim.org/) to obtain target genes related to RA disease.

### Identification for Candidate Targets of SJT in RA Therapy

Candidate targets of SJT in RA therapy were predicted by integrating the drug-target genes of SJT and disease-targets of RA. Venn diagram was drawn by Venn diagram package and the overlapping genes were screened out, which stand for the candidate targets of SJT against RA.

### Network Construction

The protein-protein interaction (PPI) regulation network was constructed through the String database (https://string-db.org/) to explain the complicated association among the compounds and target genes. The minimum required interaction score was set to 0.97. A drug-active ingredient-target network was constructed using Cytoscape 3.7.2 software (http://www.cytoscape.org) by integrating the target genes for RA disease, the active ingredients in SJT, and their corresponding targets.

### GO Analysis and KEGG Pathway Enrichment Analysis

The target genes identified were input into David v 6.8 Database for Annotation and Visualization, Integrated Discovery (https://david.ncifcrf.gov) for Gene Ontology (GO) biological process enrichment, and Kyoto Encyclopedia of Genes and Genomes (KEGG) pathway enrichment analysis. The analysis of GO and KEGG was to demonstrate the major biological processes and potential molecular mechanisms of SJT against RA.

### Statistical Analysis

Data were analyzed with SPSS 22.0 software and results were expressed as mean ± S.D. Differences between the two groups were assessed by one-way analysis of variance (ANOVA), with *p* < 0.05 being considered to be significantly different.

## Results

### Chemical Profile of SJT Determined by UHPLC-QTOF-MS

**FIGURE 2:**
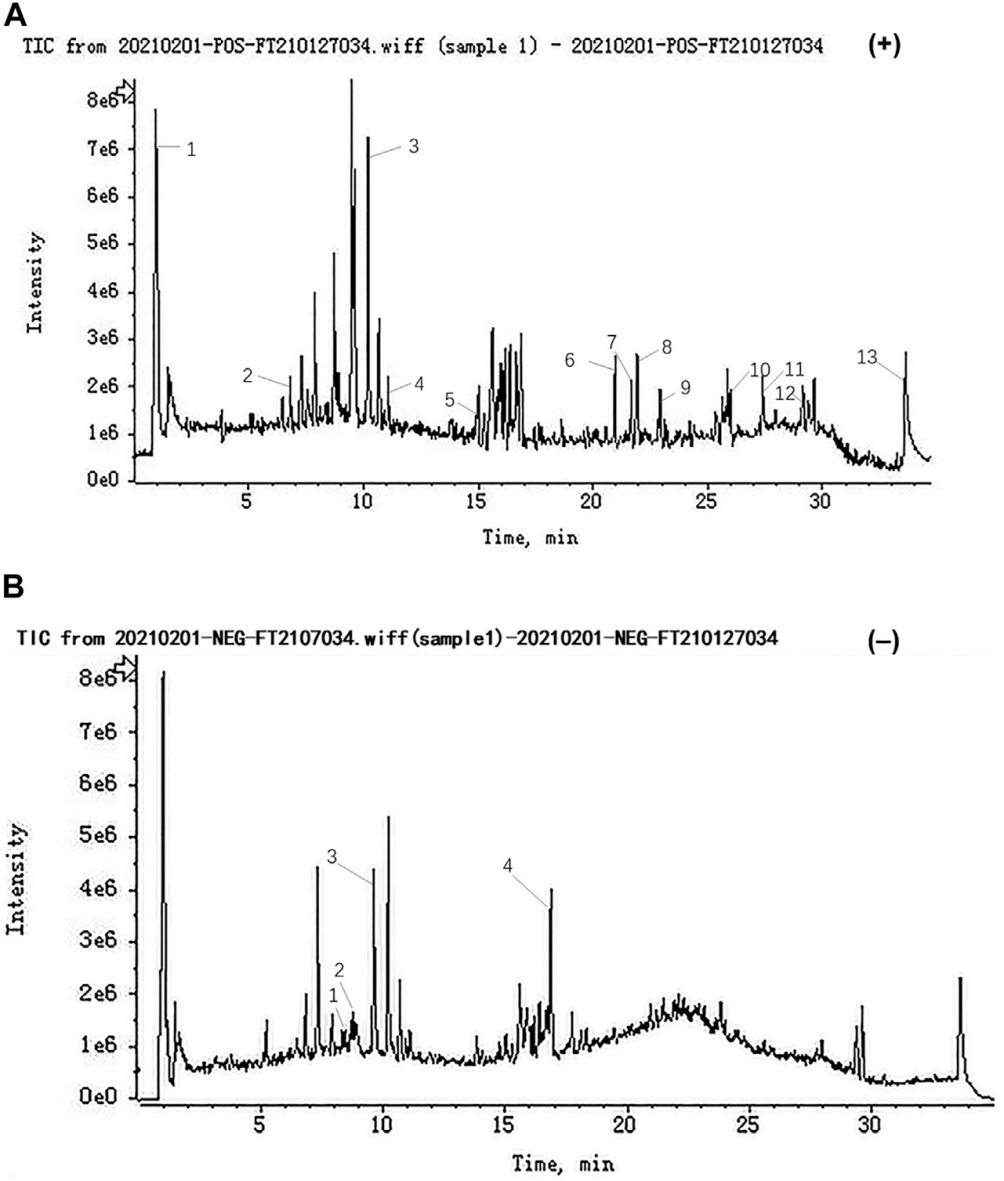
Representative base peak chromatogram (BPC) of SJT in the positive and negative ions mode, respectively. **(A)** Positive ions mode: (1) Betaine, (2) Albflorin, (3) D-Tetrandrine, (4) Ononin, (5) Bergapten, (6) Ligustilide, (7) 3-butyl-1(3H)-Isobenzofuranone, (8) 3-butylidene-1(3H)-Isobenzofuranone, (9) Columbianetin, (10) Levistilide A, (11) Stigmasterol, (12) Glycerin fatty acid ester, (13) Dioctyl phthalate, and **(B)** negative ions mode: (1) Rutin, (2) Liquiritin, (3) Quercitrin, (4) Glycyrrhizic acid.

In our study, the compounds of SJT were rapidly identified by UHPLC-QTOF-MS analysis. As a result, a total of 17 major compounds were identified, including betaine, albiflorin, D-tetrandrine, ononin, bergapten, ligustilide, 3-butyl-1(3H)-isobenzofuranone, 3-butylidene-1(3H)-isobenzofuranone, columbianetin, levistilide A, stigmasterol, glycerin fatty acid ester, dioctyl phthalate, rutin, liquiritin, quercitrin, glycyrrhizic acid **(** Figures 2A,B **)**.

### SJT Alleviated Systemic Symptoms Score and Arthritis Index in AIA Rats

**FIGURE 3:**
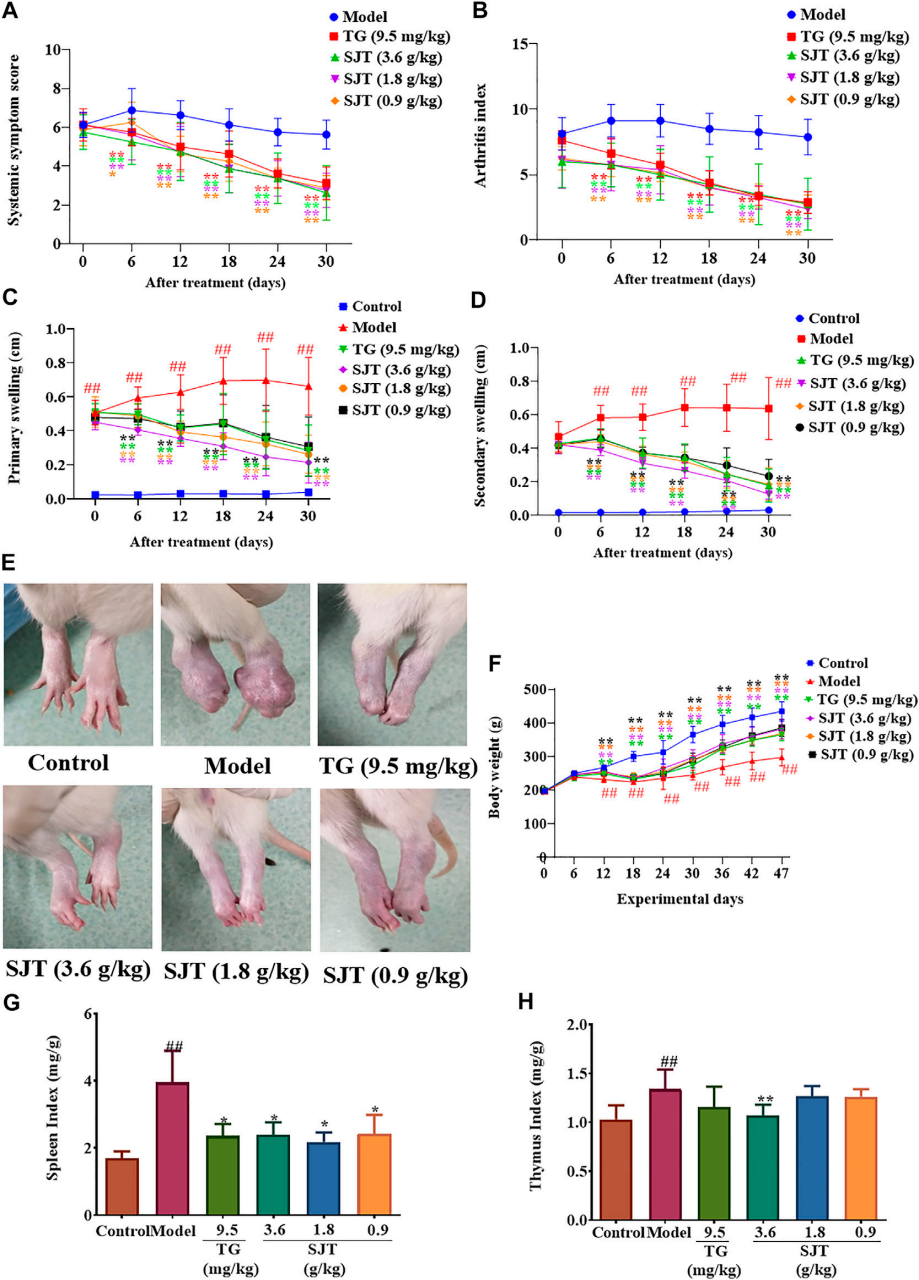
SJT suppressed rheumatoid arthritis in AIA rats. AIA rat model was established by inoculating with the complete Freund’s adjuvant (CFA) in the right hind paw. From the 17th day onward, rats were grouped and treated with or without SJT (0.9, 1.8, 3.6 g/kg) or tripterygium glycoside (TG, 9.5 mg/kg) for 30 days continuously. The rats were regularly analyzed for body weight and paw swelling, and sacrificed after drug treatment. **(A)** Arthritis index was evaluated by the 5-point ordinal scale scoring system. **(B)** Systemic symptoms scores were evaluated according to the systemic symptom score table. **(C)** Paw swelling evaluation in the primary side of AIA rats. **(D)** Paw swelling evaluation in the secondary side of AIA rats. The paw swelling was calculated by subtracting the initial (day 0) paw thickness. **(E)** Representation for paws in the secondary side of AIA rats with or without SJT treatment for 30 days **(F)** Body weights of AIA rats. Organ indexes of the spleen **(G)** and thymus **(H)** of AIA rats with or without SJT treatment for 30 days. All data were expressed as mean ± S.D. vs. model, *p < 0.05, **p < 0.01; vs. normal, #*p* < 0.05, ^##^ *p* < 0.01.

The arthritis index and systemic symptoms score are two important parameters for determining the anti-RA function of drugs, of which the arthritis index is reflecting the inflammatory response of joints, while the systemic symptoms score is referring to the inflammatory response of the whole body. In our study, SJT (3.6, 1.8, 0.9 g/kg) and TG (9.5 mg/kg) significantly reduced the systemic symptoms score (Figure 3A) and arthritis index (Figure 3B) in a dose-dependent and time-dependent manner. In the clinic, the SJT dosage of 0.071 g/kg/day is commonly recommended used in humans, which converted into rats is 0.45 g/kg. Considering the higher rate of metabolism in rodents, we chose 0.9, 1.8, 3.6 g/kg as our testing dosages in rats. As a matter of fact, according to the extraction yield of SJT, the dosages of extract were 144, 288, 576 mg/kg, which were partly consistent with the referring dose range of 100–200 mg/kg recommended in “best practice in research” (Heinrich et al., 2020).

### SJT Suppressed Paw Swelling in AIA Rats

Paw swelling is a classic character in the AIA model, and reducing paw-swelling is an important parameter to determine a remedy possessing an anti-RA effect. In our study, SJT suppressed paw swelling of AIA rats on the primary (Figure 3C) and secondary sides (Figure 3D) in a dose-dependent and time-dependent manner, showing that SJT had the capability of anti-RA effect. The paws photos also showed the strong suppression of SJT against arthritis (Figure 3E).

### SJT Alleviated the Influence of Bodyweight and Organ Index of Rats Induced by CFA

RA is a kind of chronic systemic disease with a long time lasting. Patients harboring RA disease always experience extraarticular changes and over-response of immunity, resulting in body weight loss and immune organ augment (Summers et al., 2008; Lemmey et al., 2016). In this study, the effects of SJT on body weight and immune organ index were measured accordingly. As shown in Figure 3F, compared with the normal rats, the model rats (CFA-injected only) got a significant bodyweight loss. However, after treatment with SJT or TG, the bodyweight losses of rats were significantly alleviated, in a good time-dependent manner. Furthermore, SJT showed good attenuation on the augment of immune organs induced by CFA (Figures 3G,H).

### SJT Attenuated Inflammation and Bone Lesion of Ankle Joints in AIA Rats

**FIGURE 4:**
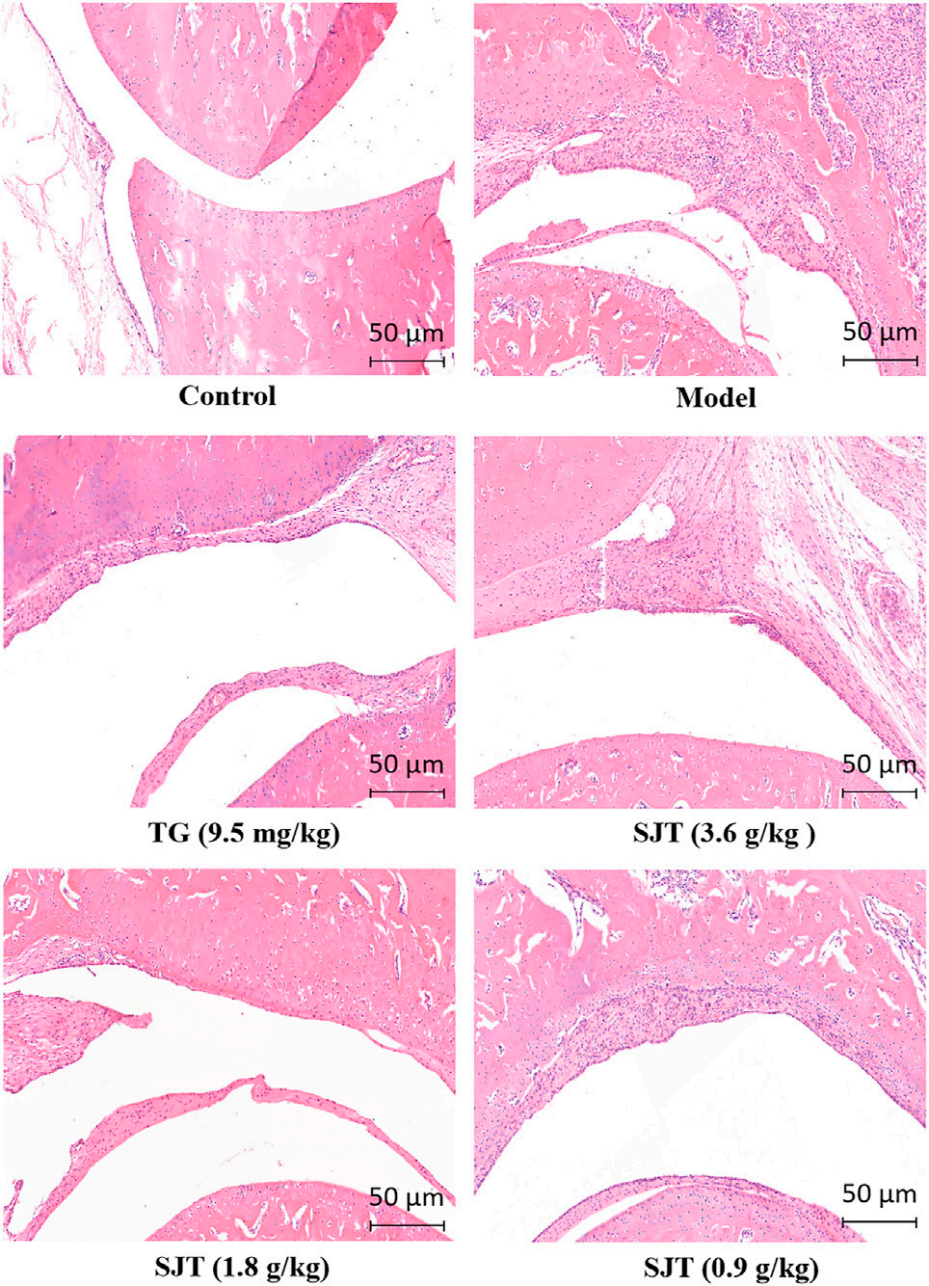
Effect of SJT on histopathological change of paws in AIA rats. Hematoxylin-eosin (HE) staining was used to detect the histopathological change of the second joints of AIA rats after SJT treatment for 30 days. The histopathological change was determined under a light microscope and images were taken. Magnification, 100 ×.

Furthermore, the effect of SJT on the inflammation and bone lesion (secondary side) induced by CFA was evaluated by hematoxylin-eosin staining. As shown in Figure 4, comparing with the normal group, pathological changes of ankle joints were observable in model group rats. CFA induced massive inflammatory cells infiltrating into the joint cavity, which resulted in synovial hyperplasia, cartilage, and bone being eroded severely. After SJT treatment for 30 days, the inflammatory cells infiltrating into the joint cavity significantly decreased, and cartilage surface, bone erosion, joints degradation were reduced, indicating that SJT strongly inhibited inflammation and bone lesion in the joints of AIA rats.

### SJT Reduced Inflammatory Cytokines in Serum of AIA Rats

**FIGURE 5:**
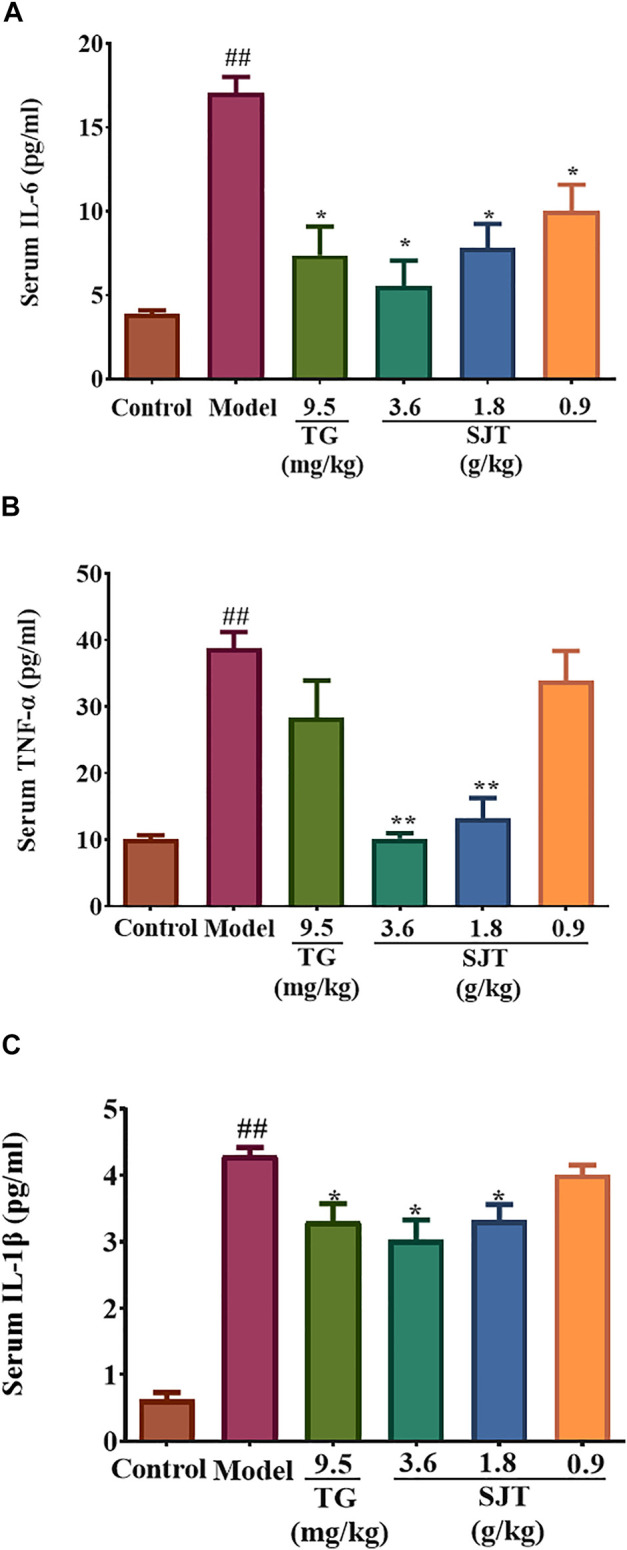
Effect of SJT on pro-inflammatory cytokines in the serum of AIA rats. The levels of IL-6 **(A)**, TNF-α **(B)**, and IL-1β **(C)** were determined by ELISA assay. Data were expressed as mean ± S.D. vs. model, *p < 0.05, **p < 0.01; vs. normal, ^#^ *p* < 0.05, ^##^ *p* < 0.01.

ELISA assay was used to detect the effect of SJT on the production of proinflammatory cytokines in the serum of AIA rats, including TNF-α, IL-1β, and IL-6. The results showed that CFA induced high levels of TNF-α, IL-1β, and IL-6, while SJT (3.6, 1.8, 0.9 g/kg) treatment remarkably reduced the high levels of these cytokines, suggesting that SJT significantly alleviated the inflammation response in AIA rats (Figure 5).

### Collection for Compounds and Target Information of SJT

SJT was composed of PL, LS, AS, AP, ST, SG, GU, SS, CS, AB, FP, TC, CC, TS. A total of 208 SJT active ingredients were obtained by searching the TCMSP database, among which the ingredients of TSM were collected by literature searching. All of the active ingredients conformed to the ADME standard (OB ≥ 30%, DL ≥ 0.18). The numbers of compounds from PL, LS, AS, AP, ST, SG, GU, SS, CS, AB, FP, TC, CC, TS were 13, 7, 2, 9, 3, 15, 92, 15, 10, 20, 1, 2, 7, 12, respectively. Based on the results, a total of 2,748 targets related to the bioactive components were picked out by searching the TCMSP database. Then, the Uniprot database was used to correct the target names. The numbers of targets for PL, LS, AS, AP, ST, SG, GU, SS, CS, AB, FP, TC, CC, TS were 95, 31, 52, 55, 31, 22, 1,257, 177, 48, 363, 13, 139, 54, 411, respectively. The reappeared targets were deleted, and a total of 445 targets was obtained finally. Bioactive compounds and related target information in each herb are shown in Supplementary Table S1.

### Collection for Disease Targets

A total of 4,465 genes related to RA disease were obtained by searching Genecards and OMIM database. According to the score of relevance, the top 30 genes were screen out. As shown in Table 1, the top 10 “high response” genes were IL6, HLA-DRB1, IL10, PTPN22, TNF, STAT4, MIF, CTLA4, PADI4, TNFRSF1A, and so on.

**TABLE 1:** Top 30 genes related with RA disease.

No.	Gene symbol	Description	Relevance score
1	IL6	Interleukin 6	110.06
2	HLA-DRB1	Major histocompatibility complex, class II, DR beta 1	105.85
3	IL10	Interleukin 10	91.97
4	PTPN22	Protein tyrosine phosphatase non-receptor type 22	81.69
5	TNF	Tumor necrosis factor	80.24
6	STAT4	Signal transducer and activator of transcription 4	72.84
7	MIF	Macrophage migration inhibitory factor	71.94
8	CTLA4	Cytotoxic t-lymphocyte associated protein 4	69.86
9	PADI4	Peptidyl arginine deiminase 4	68.66
10	TNFRSF1A	TNF receptor superfamily member 1A	67.56
11	IL2RA	Interleukin 2 receptor subunit alpha	67.24
12	LTA	Lymphotoxin alpha	66.19
13	TLR4	Toll like receptor 4	66.02
14	HLA-B	Major histocompatibility complex, class I, B	64.21
15	HLA-DQB1	Major histocompatibility complex, class II, DQ beta 1	62.58
16	IL2RB	Interleukin 2 receptor subunit beta	60.47
17	LACC1	Laccase domain containing 1	59.67
18	CCR6	C-C motif chemokine receptor 6	59.12
19	HLA-DQA1	Major histocompatibility complex, class II, DQ alpha 1	58.55
20	IL1B	Interleukin 1 Beta	56.17
21	IRF5	Interferon regulatory factor 5	55.42
22	CIITA	Class II major histocompatibility complex transactivator	53.77
23	ACP5	Acid Phosphatase 5, tartrate resistant	52.54
24	CRP	C-reactive protein	50.56
25	IL23R	Interleukin 23 receptor	49.24
26	NLRP1	NLR family pyrin domain containing 1	49.05
27	COL2A1	Collagen type II alpha 1 chain	48.62
28	FAS	Fas cell surface death receptor	48.51
29	MMP3	Matrix metallopeptidase 3	48.34
30	IL17A	Interleukin 17A	47.86

### Prediction for Candidate Targets of SJT in Anti-RA

**FIGURE 6:**
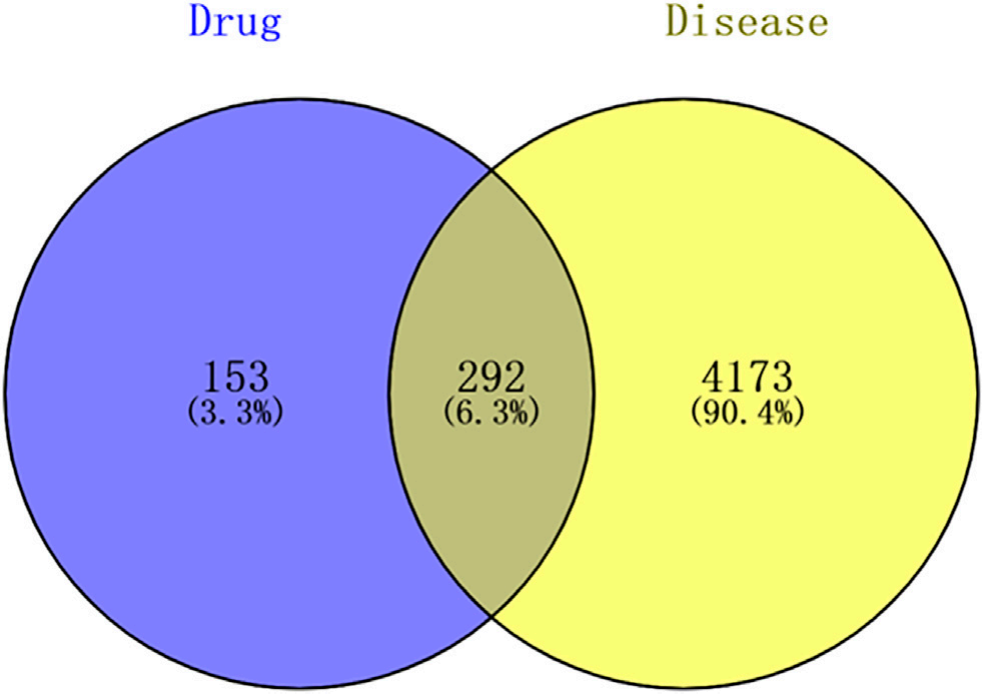
Venn diagram for the predicted targets of SJT against RA. The 292 candidate targets of SJT in RA therapy were predicted by integrating the 445 drug-target genes of SJT and 4,465 disease targets of RA.

The SJT target genes were intersected with RA disease targets to obtain the potential targets of SJT in RA therapy. The result showed that 292 overlapped genes were identified by matching the target genes of SJT with the therapeutic target genes of RA (Figure 6 and Supplementary Table S2), suggesting that SJT probably performs the function of anti-RA *via* modulating the 292 genes. The numbers of overlapping targets of PL, LS, AS, AP, ST, SG, GU, SS, CS, AB, FP, TC, CC, TS were 49, 15, 30, 19,22, 4, 145, 43, 24, 131, 2,104, 16, 6, respectively. The distribution of overlapping genes in each herb is shown in Supplementary Table S3.

### Network Construction of “SJT-Component-Target-RA”

**FIGURE 7:**
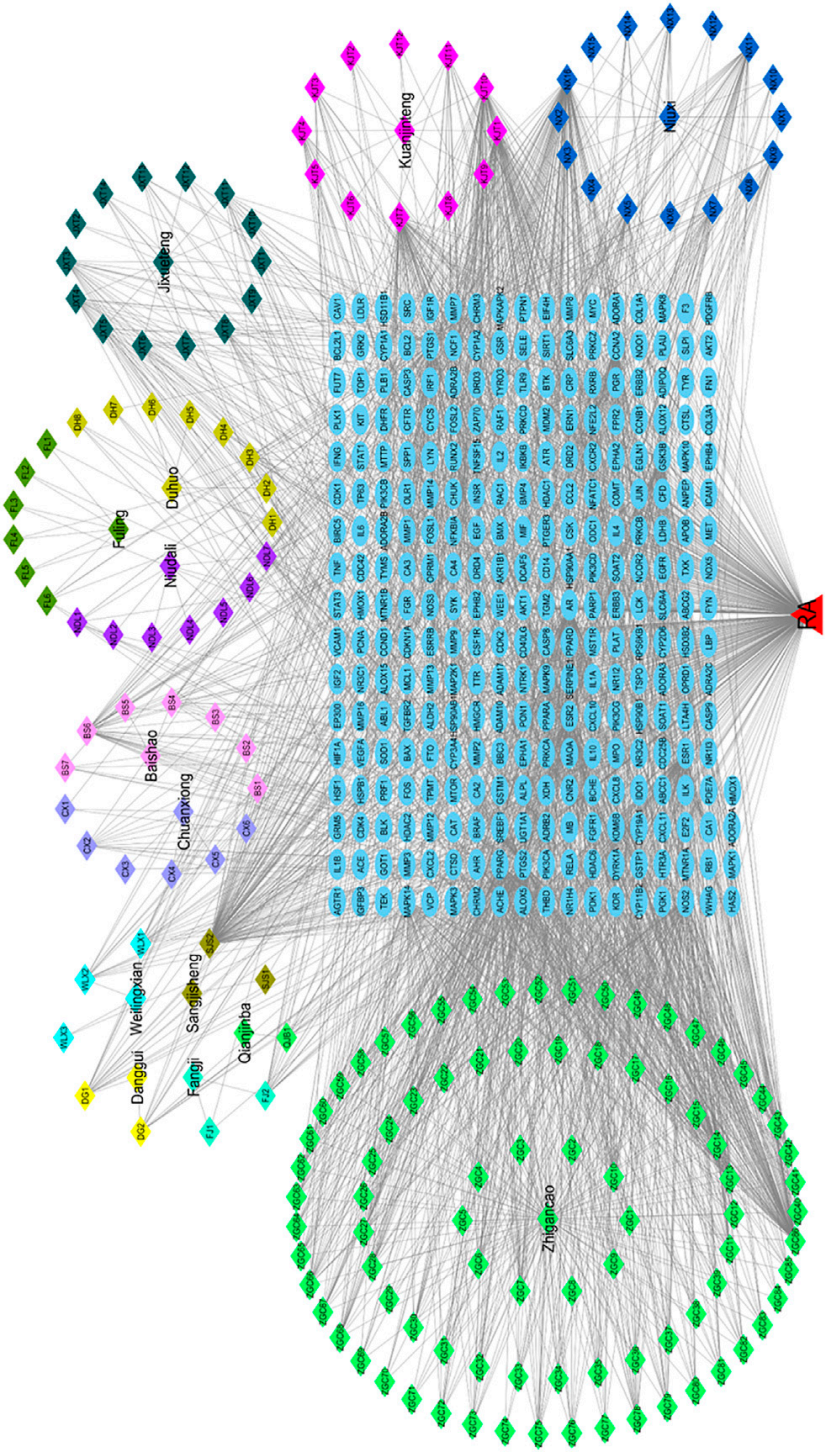
Drug-active ingredient-target network diagram. The polygon with different colors represented herbs and corresponding ingredients in the prescription of Shuji tablet. The blue ovals in the middle were the 292 collective target genes.

Cytoscape 3.7.2 software was employed to build up the “drug-component-target-disease” network. The potential active components and overlapped targets of SJT and RA were input into the system, and the “SJT-component-target-RA” network was constructed by connecting to the predicted targets. The results are shown in Figure 7.

### PPI Network Construction and Core Target Screeing

**FIGURE 8:**
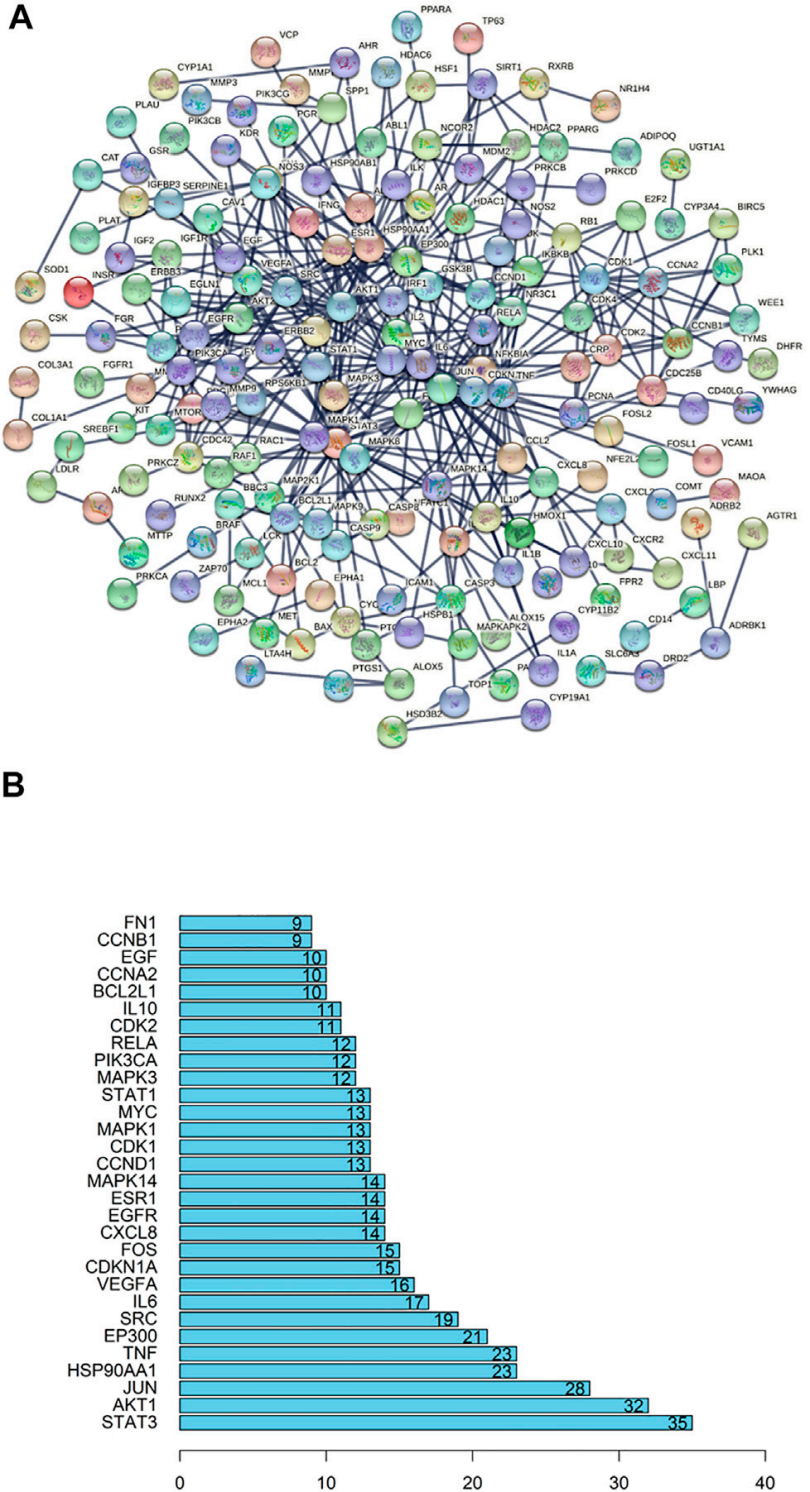
PPI (protein-protein interaction) network. **(A)** The collective genes were screened out and combined for constructing a PPI network. The nodes represented targets, and the connected lines represented interactions between different target proteins. **(B)** Bar chart for numbers of target proteins connecting with other target proteins. Data were showing the top 30 targets harboring the most quantity of connections with other targets.

Basing on the overlapped targets of SJT and RA, the protein-protein interaction (PPI) network was established by the String database (http://www.string-db.org). As shown in Figure 8A, there were 292 nodes and 467 edges in the network diagram and the average node degree was 3.2. The top 30 core genes were selected out, which had a node degree greater than 9 (Figure 8B **)**. The results showed that key targets probably were STAT3, AKT1, JUN, HSP90AA1, TNF, which possessed more connections than other genes.

### GO Analysis and KEGG Pathway Enrichment Analysis

**FIGURE 9:**
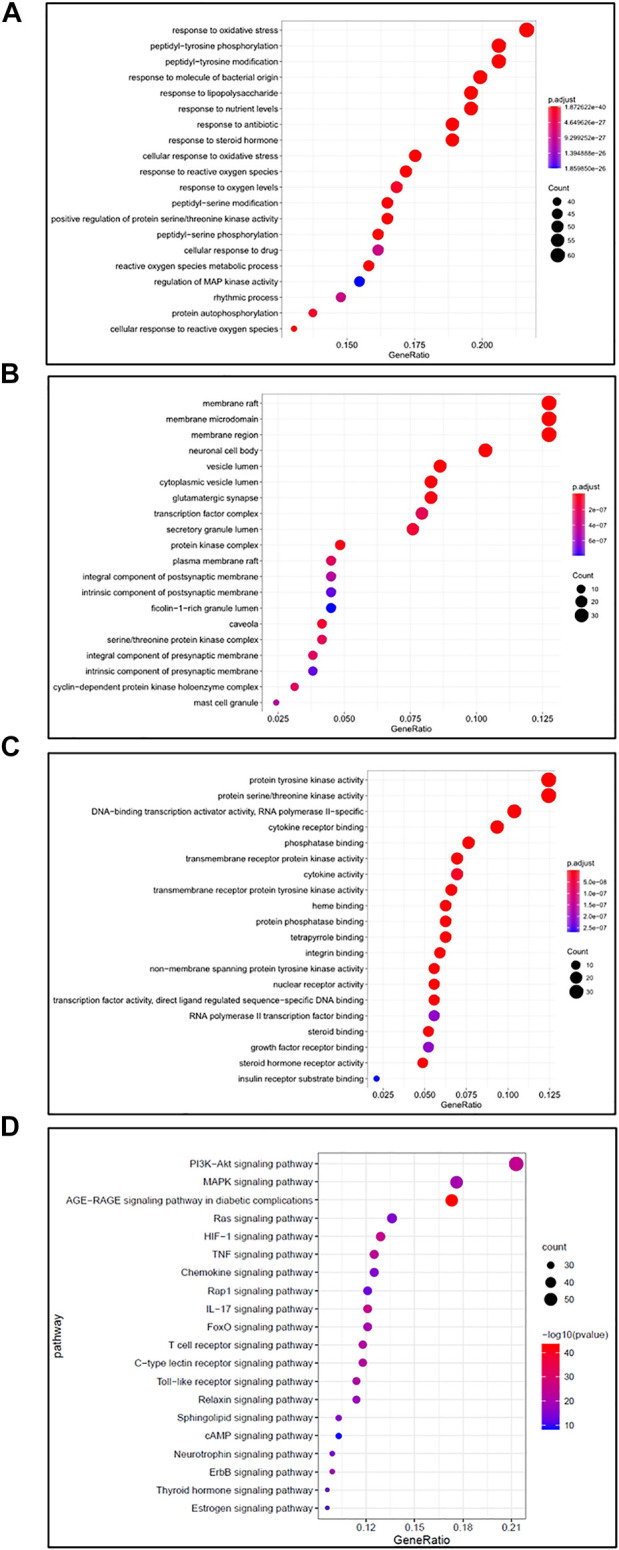
GO and KEGG pathway enrichment analysis. **(A–C)** Dot plot showed the GO analysis for SJT in anti-RA, including **(A)** biological process (BP), **(B)** cellular component (CC), **(C)** molecular function (MF). **(D)** KEGG pathway enrichment analysis for SJT in anti-RA. Dot plot showed the top 20 signaling pathways related to SJT in RA treatment.

The David v 6.8 Database (https://david.ncifcrf.gov) was applied for GO and KEGG enrichment analysis, aiming to understand the biological process (BP), cellular component (CC), molecular function (MF), and potential signaling pathways, which were involved in the anti-RA function of SJT. As a result, the numbers of BP, MF, and CC of SJT against RA were 3,053, 210, and 115 **(** Supplementary Table S4), respectively, and the top 20 GO analysis of BP (Figure 9A), CC (Figure 9B), MF (Figure 9C) were represented as graphical bubbles. Furthermore, 45 signaling pathways were identified through KEGG pathway enrichment analysis (Supplementary Table S5), and the top 20 signaling pathways were represented as a bar graph (Figure 9D), among which, PI3K-Akt pathway, MAPK pathway, Ras pathway, HIF-1 pathway, TNF pathway, Chemokine pathway, IL-17 pathway, FoxO pathway, Rap1 pathway, AGE-RAGE signaling pathway in diabetic complications had been proved associated with the treatment of RA.

## Discussion

RA is a kind of chronic disease lasting for a long course, and there is no drug used in the clinic that can cure RA successfully. RA patients would not only experience arthrophlogosis such as joints swelling and stiffness, but also other diseases with extraarticular symptoms or systemic manifestations, such as rheumatoid nodules, vasculitis, cardiovascular disease, and so on (Hochberg et al., 2008). Early diagnosis and early treatment are the key strategies to improve the outcome of RA therapy. However, patients mostly are diagnosed with RA in the advanced phase, so they miss the best chance for therapy. RA is a systemic disease owing to the feature of disordered multiple-biological processes, -signaling pathways, and -target proteins. Hence, a remedy with comprehensive treatment is demanded by targeting multiple biological processes, -mechanisms, and -targets. It is a good strategy to develop a new therapeutic method basing on traditional remedies or ethnological medicine to overcome RA disease. Furthermore, ameliorating systemic manifestations by inhibiting inflammation response can reduce the mortality of RA, and is recognized to be a common therapeutic approach in the clinic.

Traditional Chinese medicine (TCM) possesses a long history of application and shows good potency of RA therapy in the clinic. Precious studies showed that several traditional Chinese herbal medicines such as Wangbi tablet (Wang et al., 2020) and Wutou decoction (Guo et al., 2017) had a good effect in treating RA. The prescriptions were discovered to modulate the imbalance of the whole body and ameliorate local symptoms accordingly. Especially, Qing-Luo-Yin not only shows an anti-rheumatic effect but also antagonizes the toxicity and side effect induced by methotrexate (Zuo et al., 2018). SJT, a traditional Chinese medicine prescription, was approved using in the clinic for treating lumbago and leg pain. SJT was also found to alleviate the symptom of RA patients during clinical practice. However, the experimental data against RA disease is still limited. In this study, we evaluated the anti-RA effect of SJT on AIA rats, and the results showed that SJT could dose-dependently and time-dependently ameliorate arthritis symptoms, whole-body inflammation response, and also reduce the levels of serum cytokines relating with pro-inflammation, all of which were consistent with what we have observed in clinical practice. The results of UHPLC-QTOF-MS showed that Betaine, Albflorin, D-Tetrandrine, Ononin, Bergapten, Ligustilide, 3-butyl-1(3H)-Isobenzofuranone, 3-butylidene-1(3H)-Isobenzofuranone, Columbianetin, Levistilide A, Stigmasterol, Glycerin fatty acid ester, Dioctyl phthalate, Rutin, Liquiritin, Quercitrin, Glycyrrhizic acid were the major ingredients in SJT. Among them, D-Tetrandrine (Li et al., 2018), Ononin (Meng et al., 2021), Stigmasterol (Ahmad et al., 2020), Rutin (Sun et al., 2017), Liquiritin (Zhai et al., 2019) were reported to have an anti-RA function in previous research, which suggested that SJT had the potential property of treating RA.

With the rapid development of bioinformatics technology, network pharmacology is usually employed to speed up the progress of drug research and has become a promising approach for drug discovery and development, especially for TCM study due to its complicated ingredients. According to the new guidance, the network pharmacology evaluation should be conducted in three aspects: reliability, standardization, and rationality (Li et al., 2021). Actually, in our study, to ensure the reliability of TCM network pharmacology, all the data were collected from the TCMSP database, which recorded 499 common traditional Chinese medicines. A total of 208 active ingredients were obtained from 14 herbs of SJT. After that, the databases of PubChem and Uniprot were used to correct the compound names and molecular structures to make sure that the relevant data can be traced based on our description. Simultaneously, setting parameters of “DL ≥ 0.18 and OB ≥ 30%”, “The minimum required interaction score being 0.97”, and “hiding disconnected nodes in the network” to make sure that data were completed and consistent with our research objective. The top five herbs which harbored the most quantity of ingredients were PL, SG, GU, AB, TS, with compound numbers of 13, 15, 92, 20, 12, respectively. However, the distribution of overlapping genes in each herb showed a different result. PL, GU, SS, AB, TC were the top five herbs possessing the most quantity of overlapping genes, with gene numbers of 49, 145, 43, 131, 104, respectively. Probably, the reason was that some compounds from the herbs which contained few ingredients would have more targets associated with RA therapy. For instance, TC had only two active compounds but 104 genes related to RA.

KEGG pathway enrichment analysis showed that there were 45 signaling pathways directly linked to RA, indicating that SJT might modulate these signaling pathways against RA. The top 10 signaling pathways firmly related to RA therapy were PI3K-Akt pathway, MAPK pathway, AGE-RAGE signaling pathway in diabetic complications, Ras pathway, HIF-1 pathway, TNF pathway, Chemokine pathway, IL-17 pathway, FoxO pathway, Rap1 pathway. Most of these signaling pathways had been proved associated with the occurrence and development of RA. PI3K-AKT signaling pathway is an intracellular signaling pathway that regulates the cell proliferation and cell cycle process and resists apoptosis, angiogenesis, autophagy (Johnson et al., 2010; Xia et al., 2015; Manfredi et al., 2015). Regulating the metabolism of chondrocytes *via* PI3K/AKT pathway would affect the disease progression in RA rats (Feng et al., 2018). Mitogen-activated protein kinase (MAPK) signaling is a fundamental pathway in cell biology and plays an important role in the pathophysiological process of human diseases (Yuan et al., 2020). Activation of p38 MAPK contributes to almost all RA-related pathologies, including synovial inflammation, damage of cartilage and bone, and angiogenesis (Schett et al., 2008). Inhibiting MAPK signaling pathways would reduce RA-associated ROS accumulation, leading to suppression of inflammation and cell proliferation of synovial cells, as well as mitigation of angiogenesis (Yang et al., 2018). Ras superfamily of GTPases plays an important role in the immune system of the human body (Zayoud et al., 2017). T cells highly expressing K-Ras would induce autoimmunity, which was mediated by citrullinated vimentin-derived peptide, a pathogenic autoantigen in RA (Weaver et al., 2007; Singh et al., 2009). Blocking the Ras signaling pathway had been proved to be a promising therapeutic approach for RA (Zayoud et al., 2017). Hypoxia is one of the major characteristics of RA synovium. Hypoxia-inducible factors (HIFs) are transcription factors that can promote glycolysis to produce energy *via* enhancing gene expressions of glucose transporters and glycolytic enzymes (Fearon et al., 2016). RA synovium cells within the hypoxic environment can survive by an adaptive mechanism mediated by the HIF-1 pathway (Harris et al., 2002). HIF-1ɑ enhances the catalytic activity of lactate dehydrogenase A (LDHA), resulting in the hyper-acidic microenvironment, which would promote synovium cell proliferation and invasion (Fearon et al., 2016). Overexpression of HIF-1ɑ increases the production of IFN-γ and IL-17 (Larsen et al., 2012), and enhances the effects of IL-1β and TNF on angiogenesis and invasion in RA (Li et al., 2013). Tumor necrosis factor (TNF) and its receptors, two kinds of transmembrane proteins of immune cells, play a key role in the inflammation response and the development of RA (Blüml et al., 2012). It is a kind of pleiotropic cytokine that enhances synovial proliferation, produces prostaglandins and metalloproteinases, as well as regulates other proinflammatory cytokines (Taylor et al., 2009). Hence, TNF is considered a useful therapeutic target for RA therapy. Agents targeting TNF have been proved to be effective in treating RA in the clinic (Taylor et al., 2009). Rheumatoid synovial fibroblasts secrete a lot of matrix-degrading metalloproteinases (MMPs), which lead to tissue damage by proteolytic degradation of collagens and proteoglycans. IL-17 can modulate MMP-1 and its inhibitor TIMP-1 to regulate the progression of RA (Chabaud et al., 2000). Cysteine-rich protein 61 (CYR-61) plays a key role in the pathogenesis of RA, and activation of Fox signaling would induce the secretion of CYR-61 in rheumatoid synovial fibroblasts (Kok et al., 2013). Therefore, targeting Fox signaling pathway would be also an important therapeutic regimen for RA disease. The chemokine signaling pathway is involved in the progression of RA by mediating leukocyte extravasation and is considered to be a good target pathway for RA therapy. As reported, blocking C-C motif chemokine receptor 1 (CCR 1) was proved to be a promising therapeutic approach against RA (Szekanecz et al., 2010). As a small G protein in the Ras superfamily, Ras-proximate-1 (Rap 1) plays an important role in modulating intracellular signaling pathways and stimulating T cells (Remans et al., 2006). Rap1 signaling regulates the production of reactive oxygen species (ROS) and is associated with oxidative stress of T cells infiltrated in the RA synovium (Remans et al., 2004). Advanced glycation end products (AGEs) are generated as a result of oxidative stress during chronic inflammation and sever as a measurement of cumulative inflammation in RA patients (de Groot et al., 2011).

As expected in our study, SJT reduced the levels of IL-1β, IL-6, and TNF-α in the serum of AIA rats, indicating SJT could suppress RA by modulating the inflammation-related pathways, which were also predicted in the follow-up study by network pharmacological approach. However, the detailed modulated mechanism of SJT against RA needs further investigation, including the modulation on more detailed inflammatory pathways, metabolism pathways, and immune pathways related to RA. Furthermore, the network pharmacology analysis should be done by more a rational and normative process, and the parameters of accuracy, precision, recall should be considered sufficiently. Employing a professional platform such as the integrative TCM network pharmacology platform (Zhang et al., 2013) is necessary to ensure all data can be traced easily.

## Conclusion

Our findings demonstrated that SJT strongly alleviated rheumatoid arthritis and reduced the secretion of IL-6, IL-1β, TNF-α of AIA rats. Network pharmacological analysis showed that the key targets of SJT in the treating RA probably were STAT3, AKT1, JUN, HSP90AA1, TNF, and the potential mechanism would be associated with modulation on PI3K-Akt, MAPK, Ras, AGE-RAGE, HIF-1, TNF, chemokine, IL-17, FoxO, Rap 1 signaling pathways. Our study provides evidence for Shuji tablet in RA therapy and would expand the usage of Shuji tablet in the clinic.

## Data Availability

The raw data supporting the conclusion of this article will be made available by the authors, without undue reservation, to any qualified researcher.

## Supplementary Material

Supplementary Table S1 Compounds from the herbals of Shuji tablet (SJT) based on the network approach.

Supplementary Table S2 The overlapping genes of Shuji tablet (SJT) and rheumatoid arthritis (RA).

Supplementary Table S3 The distribution of overlapping genes in each herb.

Supplementary Table S4 GO analysis for biological process (BP), molecular function (MF), cellular component (CC).

Supplementary Table S5 Signaling pathways identified *via* KEGG pathway enrichment analysis.
